# Serum 25(OH)vitamin D level and Its correlation with bone mineral density in girls with Adolescent Idiopathic Scoliosis (AIS)

**DOI:** 10.1186/1748-7161-10-S1-O7

**Published:** 2015-01-19

**Authors:** Tsz Ping Lam, Bobby Kin Wah Ng, Kwong Man Lee, Alec Lik Hang Hung, Elisa Man Shan Tam, Franco Tsz Fung Cheung, Echo Ka Ling Tsang, Jack Chun Yiu Cheng

**Affiliations:** 1 Department of Orthopaedics and Traumatology, The Chinese University of Hong Kong, Hong Kong; 2 Lee Hysan Clinical Research Laboratories, The Chinese University of Hong Kong, Shatin, Hong Kong; 3 Joint Scoliosis Research Center of the Chinese University of Hong Kong and Nanjing University, China

## Objectives

AIS is associated with both low bone mass and elevated serum bone alkaline phosphatase. It was also reported the prevalence of AIS was positively correlated with the latitude of the geographical region. These specific features were compatible with the presence of either Vit-D insufficiency or abnormal physiology with Vit-D. It is important to evaluate these potentially treatable conditions regarding their roles in the etiopathogenesis of AIS. The objectives of this case-control study were to evaluate Vit-D status and its correlation with areal bone mineral density (aBMD) in AIS subjects and normal controls.

## Material and methods

212 AIS girls and 183 age and gender-matched normal controls (mean age 12.9, SD=0.6 and 12.9, SD=0.5 years old respectively, p=0.506) were recruited in summer and winter. Serum 25(OH)Vit-D was measured with Liquid-chromatography Tandem-mass-spectroscopy and aBMD at femoral necks was measured with Dual-energy X-ray Absorptiometry.

## Results

The mean aBMD at the right and left side for AIS subjects were 0.751 (SD=0.109), 0.744 (SD=0.107), and that for controls were 0.785 (SD=0.114) and 0.785 (SD=0.116) gm/cm2 respectively. The mean 25(OH)Vit-D levels for AIS and controls were 41.6 (SD=14.4) and 39.5 (SD=11.5) nmol/L respectively (p=0.103). Using multivariate linear regression model to adjust for age, body weight, armspan, season, physical activity and dietary calcium intake levels, the p-value on the correlation between aBMD and 25(OH)Vit-D level for the right and left side for controls were 0.055 and 0.047, and that for AIS were 0.804 and 0.466 respectively.

## Conclusions

AIS and Control group had suboptimal 25(OH)Vit-D levels. The positive correlation between 25(OH)Vit-D and aBMD seen in controls was not present in AIS subjects, thus indicating the possibility of Vit-D resistance in AIS. Whether this is responsible for osteopenia that characterizes AIS and how this is related to the etiopathogenesis of AIS warrant further studies.

## Funding source

GRF of RGC of Hong Kong (Project no: 468809 and 468411).

